# Predicting major adverse cardiovascular events in angina patients using radiomic features of pericoronary adipose tissue based on CCTA

**DOI:** 10.3389/fcvm.2024.1462451

**Published:** 2024-10-31

**Authors:** Weisheng Zhan, Yanfang Luo, Hui Luo, Zheng Zhou, Nianpei Yin, Yixin Li, Xinyi Feng, Ying Yang

**Affiliations:** ^1^ Department of Cardiology, The Affiliated Hospital of North Sichuan Medical College, Nanchong, Sichuan, China; ^2^ Department of Thoracic Surgery, Nan Chong Center Hospital, Nanchong, China

**Keywords:** coronary computed tomography angiography, major adverse cardiovascular event, pericoronary adipose tissue, angina pectoris, radiomics

## Abstract

**Objective:**

This study aims to evaluate whether radiomic features of pericoronary adipose tissue (PCAT) derived from coronary computed tomography angiography (CCTA) can better predict major adverse cardiovascular events (MACE) in patients with angina pectoris.

**Methods:**

A single-center retrospective study included 239 patients with angina pectoris who underwent coronary CT examinations. Participants were divided into MACE (*n* = 46) and non-MACE (*n* = 193) groups based on the occurrence of MACE during follow-up, and further allocated into a training cohort (*n* = 167) and a validation cohort (*n* = 72) at a 7:3 ratio. Automatic segmentation of PCAT surrounding the proximal segments of the left anterior descending artery (LAD), left circumflex coronary artery (LCX), and right coronary artery (RCA) was performed for all patients. Radiomic features of the coronary arteries were extracted, screened, and integrated while quantifying the fat attenuation index (FAI) for the three vessels. Univariate and multivariate logistic regression analyses were utilized to select clinical predictors of adverse cardiovascular events. Subsequently, machine learning techniques were employed to construct models based on FAI, clinical features, and radiomic characteristics. The predictive performance of each model was assessed and compared using receiver operating characteristic (ROC) curves, calibration plots, and decision curve analysis for clinical utility.

**Results:**

The radiomics model demonstrated superior performance in predicting MACE in patients with angina pectoris within both the training and validation cohorts, yielding areas under the curve (AUC) of 0.83 and 0.71, respectively, which significantly outperformed the FAI model (AUC = 0.71, 0.54) and the clinical model (AUC = 0.81, 0.67), with statistically significant differences in AUC (*p* < 0.05). Calibration curves for all three predictive models exhibited good fit (all *p* > 0.05). Decision curve analysis indicated that the radiomics model provided higher clinical benefit than the traditional clinical and FAI models.

**Conclusion:**

The CCTA-based PCAT radiomics model is an effective tool for predicting MACE in patients with angina pectoris, assisting clinicians in optimizing risk stratification for individual patients. The CCTA-based radiomics model significantly surpasses traditional FAI and clinical models in predicting major adverse cardiovascular events in patients with angina pectoris.

## 1 Introduction

As public health awareness increases, the incidence of coronary artery disease (CAD) has seen a decline. However, it continues to pose a threat to human safety globally (1). According to recent statistics, half of the patients diagnosed with CAD suffer from angina pectoris (2). Angina is defined as chest pain, pressure, or discomfort located behind the sternum, which is often exacerbated by exertion and/or anxiety or other emotional or psychological stresses (3, 4). It is a highly prevalent condition that seriously endangers individuals’ health. With advancing age, the prevalence of angina increases, and concurrently, the risk of cardiovascular and cerebrovascular accidents such as congestive heart failure, myocardial infarction, and malignant arrhythmias also rises (5, 6). Therefore, identifying high-risk angina patients is crucial for effective risk stratification, patient management, and rational allocation of public health resources. Currently, the monitoring and assessment of the progression of angina primarily rely on traditional clinical factors such as age, risk factors, and medication history (7, 8). For patients with worsening chest pain symptoms or increased frequency of episodes, coronary CTA, a preferred non-invasive imaging method in clinical practice, is used for further examination. Based on the results of vascular stenosis, we then assess and predict the likelihood of patients experiencing major adverse cardiovascular events (9, 10). Recent research indicates that the formation and rupture of atherosclerotic plaques are inseparably linked to inflammation within the vessel (11). When vascular inflammation occurs, pro-inflammatory factors released diffuse into the PCAT via paracrine signaling, inhibiting preadipocyte differentiation and lipid accumulation (12–14). Researchers have discovered a new sensitive biomarker of coronary artery inflammation—the fat attenuation index (FAI)—which captures the CT attenuation gradient of PCAT, thereby revealing changes in the composition of PCAT induced by vascular inflammation (10). However, the pathophysiology of coronary vascular lesions is complex, and relying solely on FAI provides only a planar calculation of changes, failing to reflect the complexity of spatial variations (15). With the application of artificial intelligence in clinical radiology, advanced radiomics technology is playing an increasingly pivotal role in the medical field. Radiomics is a technique for fine phenotypic analysis of extracted tomographic images. In essence, this technology converts images into data (12, 13). It has been widely applied in the clinical diagnosis, prognosis, and postoperative monitoring of tumors (14, 16). Recently, the utility of radiomics combined with coronary CT in the cardiovascular domain has gained increasing attention. Numerous studies have also verified the superiority of radiomic features of pericoronary adipose tissue in diagnosing myocardial infarction and differentiating coronary heart disease (17, 18). However, the role of radiomics of pericoronary adipose tissue based on CCTA in predicting major clinical events has not yet been further investigated. Therefore, our study aims to explore the role of radiomic features based on CCTA in predicting major adverse cardiovascular events in patients with angina pectoris. In addition, we aim to compare it with traditional imaging techniques and clinical methods to determine whether it can improve and optimize the predictive performance for MACE in patients with angina pectoris.

## 2 Materials and methods

### 2.1 Study population

**Figure 1:**
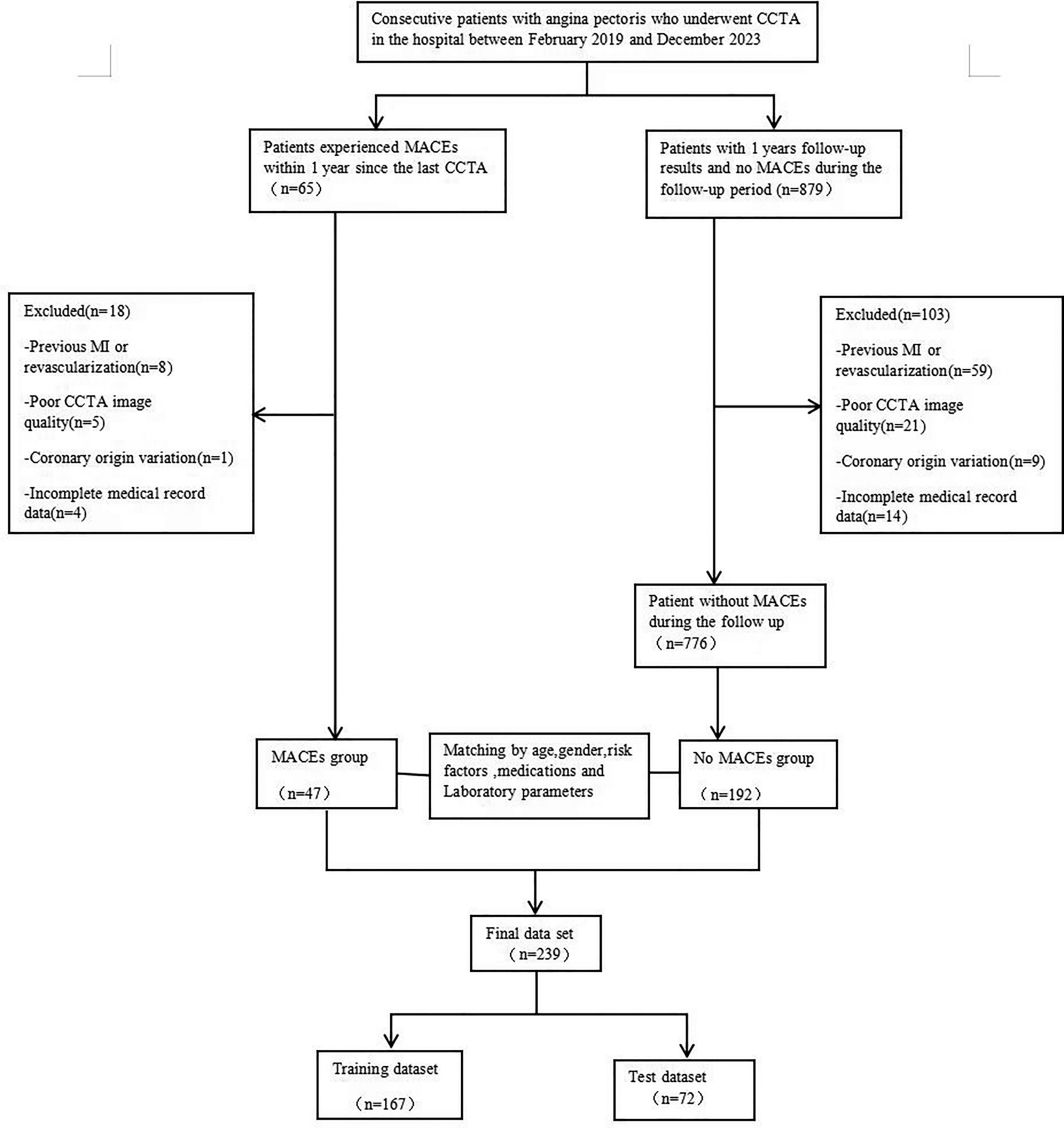
A flowchart of patient recruitment and study design. CCTA, coronary computed tomography angiography; MACE,Major adverse cardiovascular event;MI, myocardial infarction.

A retrospective collection was conducted on 239 patients with angina pectoris who underwent CCTA examinations at the Cardiac Center of the Affiliated Hospital of North Sichuan Medical College from February 2019 to December 2023. The diagnosis of angina pectoris was made by groups led independently by two cardiologists from our institution, based on medical history, physical examination, laboratory tests, and imaging studies. Exclusion criteria included a history of myocardial infarction or revascularization, poor CCTA image quality, and incomplete medical records. A detailed flowchart outlining the patient selection process and study design is provided in Figure 1. This study was approved by the Medical Ethics Committee of the Affiliated Hospital of North Sichuan Medical College (approval number: 2023ER218-1).

### 2.2 General data collection and clinical endpoints

Relevant clinical history data were collected for all patients, including demographic characteristics (gender, age, and body mass index), cardiovascular risk factors (smoking, hypertension, diabetes, hyperlipidemia, and medication history), Renal Function Measurement [Estimated glomerular filtration rate (eGFR), Serum creatinine (Scr) levels and blood urea nitrogen (BUN)] as well as laboratory and echocardiographic parameters.

The clinical endpoint was the occurrence of MACEs, defined as a composite of death, malignant arrhythmias, new-onset congestive heart failure, acute myocardial infarction, and cerebral infarction within one year.

Anti-Anginal Therapy Adjustments: During the follow-up period, modifications in anti-anginal therapy were documented for all patients. Adjustments included changes in the dosage or addition of medications such as beta-blockers, calcium channel blockers, and nitrates, based on patients’ symptoms and clinical assessments. These modifications were tracked and analyzed as part of the study.

### 2.3 CCTA acquisition

All recruited subjects underwent scanning with a 2*96-detector row CT scanner (Stellar Infinity detector, SOMATOM Force, Siemens Healthineers, Germany). The scanner operated with prospective ECG gating and tube voltage ranging from 70 to 150 kV, a rotation speed of 0.25 s, and a temporal resolution of 66 ms per segment, enabling free heart rate coronary CT. It also utilized Edge and UHR technology with a holographic photon detector that reduces electronic crosstalk between adjacent detectors, allowing the reconstruction of thin slices from a 0.6 mm acquisition layer thickness to 0.4 mm. Due to the frequent and substantial gas exchange during respiration, which can impair the field of view, patients were instructed to hold their breath as much as possible during the coronary CT scan. Additionally, patients were positioned supine, with as much of the anterior chest exposed as possible. The instrument scanned from the head downwards, capturing images from 1 cm below the tracheal bifurcation to the apex of the heart.

### 2.4 Quantification measurement of FAI

PCAT has become a new significant target for the detection of coronary artery disease, located at a radial distance from the vessel wall, approximately equivalent to the diameter of the vessel. FAI is determined by calculating the average attenuation value of the perivascular adipose tissue (i.e., the average CT value of PCAT), and to exclude non-adipose tissues, the CT value range is generally set between −190 and −30 HU in studies. In previous research, the FAI of the surrounding tissue near the proximal RCA has been found to be more representative of inflammation compared to other coronary artery fat attenuation tissues and is considered a good indicator of systemic inflammation (19, 20). Therefore, we will use software to automatically define and measure the proximal area of the RCA (10–50 mm from the RCA ostium), ensuring high standardization and repeatability. FAI for all included patients was measured using CoronaryDoc software version 6.21 (Shukun Technology Co., Ltd, Beijing, China).

### 2.5 PCAT segmentation and radiomic feature extraction

Recent studies have suggested that pericoronary adipose tissue (PCAT) surrounding coronary artery plaques may serve as a sensitive imaging biomarker for plaque vulnerability (17). To reflect the changes in coronary arteries during inflammation as comprehensively as possible, we included all three main epicardial vessels for segmentation. Based on the method by Oikonomou (20) et al., we utilized Shukun Technology software [Shukun Technology Co., Ltd, Beijing, China, Version: 6.21] to automatically track a 40 mm segment of interest around the proximal segments of the left anterior descending artery, left circumflex coronary artery, and right coronary artery. For LAD and LCX, a 40 mm segment following the left main bifurcation was analyzed. For the RCA, to avoid the influence of the aortic wall, we excluded the proximal 10 mm and focused on the segment from 10 mm downstream of the aortic root to 50 mm proximal to the RCA. Additionally, while segmenting the plaques in the corresponding coronary regions, we set the cross-sectional area of segmentation to be three times the diameter of the vessel lumen, further ensuring the comprehensiveness of the data. A total of 94 radiomic features were extracted from the PCAT surrounding each coronary artery plaque, including morphological features, first-order histogram features, and higher-order texture features, resulting in a total of 94*3 radiomic features per patient.

### 2.6 Feature selection and prediction model building

This study attempted to develop three models to determine the prediction of MACE in patients with angina pectoris; the building process for each is detailed below.

#### 2.6.1 Radiomics model

First, the acquired raw imaging data underwent z-score normalization to exclude variables with zero variance and to center the feature values at zero. The normalized radiomic features were then subjected to Mann-Whitney *U*-tests and selection. Only radiomic features with *p*-values < 0.05 were retained. Second, we calculated the correlation between features using Spearman's rank correlation coefficient and excluded radiomic features with a correlation coefficient >0.9. Third, the remaining radiomic features underwent final selection using the Least Absolute Shrinkage and Selection Operator (LASSO) algorithm, setting irrelevant feature coefficients precisely to zero while retaining non-zero coefficient features for model fitting (21). After this series of selections, we input the final radiomic features into machine learning (random forest) to construct a predictive risk model.

#### 2.6.2 Clinical model

Independent clinical predictors, including cardiovascular risk factors, laboratory, and echocardiographic parameters, etc., were initially screened using univariate Logistic regression. Variables with *P* < 0.05 in the univariate analysis were used for subsequent multivariate Logistic analysis to determine the features most correlated with MACE. Clinical features were also input into machine learning (random forest) to develop a clinical prediction model.

#### 2.6.3 FAI model

The construction of the FAI model was nearly identical to the previous two methods. The extracted FAI values around the proximal RCA were used to establish the prediction model, using the same machine learning model. To avoid overfitting and balance the limited sample size, all three ML models were calibrated using a 10-fold cross-validation method (22).

### 2.7 Statistical analysis

Statistical analyses in this study were performed using R Studio (version 4.0.3) and Python (version 0.13.2) software packages. Continuous variables were assessed using the Mann-Whitney *U*-test or Student's *t*-test. Categorical variables were compared using the chi-square test or Fisher's exact test. For continuous (quantitative) data, the Shapiro-Wilk normality test was used to determine the normality of sample data. If the data followed a normal distribution, it was presented as mean ± standard deviation (X ± S), and comparisons between two groups were made using independent sample *t*-tests; if not normally distributed, data were presented as median (25th percentile, 75th percentile), and comparisons between two groups were made using the Wilcoxon test. A two-sided *p*-value < 0.05 was considered statistically significant. Receiver operating characteristic curves were used to evaluate the efficacy of the three types of models in predicting the occurrence of MACE in patients with angina pectoris. Calibration curves were plotted to assess the consistency between the predicted probabilities by the models and the actual probabilities. Clinical utility was evaluated through decision curve analysis (DCA), which quantifies net benefit across different threshold probabilities for the three predictive models.

## 3 Results

### 3.1 General baseline information

Table 1 summarizes the clinical baseline characteristics of the 239 patients in both the training and validation sets. Among the included patients with angina pectoris, 47 (19.7%) experienced MACEs during the follow-up period: 9 (3.8%) had a new acute ischemic stroke, 10 (4.2%) developed congestive heart failure, 8 (3.3%) suffered malignant arrhythmias, and 20 (8.4%) had a new acute myocardial infarction. Statistical testing showed that clinical features were well-matched between the MACE and non-MACE groups in both the training and validation datasets.

**Table 1:** Baseline characteristics of the study population.

Characteristics	Total (*n* = 239)	No MACEs group (*n* = 192)	MACEs group (*n* = 47)	*P*-value
Clinical characteristics Age	63.95 ± 10.78	62.96 ± 10.01	67.96 ± 12.82	0.008
Smoking	0.37 ± 0.48	0.35 ± 0.48	0.47 ± 0.50	0.142
BMI	25.23 ± 2.68	25.37 ± 2.63	24.69 ± 2.85	0.060
Male gender	130 (54.39)	103 (53.65)	27 (57.45)	0.760
Hypertension	137 (57.32)	105 (54.69)	32 (68.09)	0.134
Diabetes	45 (18.83)	34 (17.71)	11 (23.40)	0.492
Baseline medications Antiplatelet	44 (18.41)	33 (17.19)	11 (23.40)	0.438
Beta−blocker	16 (6.69)	11 (5.73)	5 (10.64)	0.378
ACEI/ARB	26 (10.88)	19 (9.90)	7 (14.89)	0.468
Statin	0.17 ± 0.38	0.17 ± 0.37	0.19 ± 0.40	0.741
Lipids, mmol/L Triglycerides	1.70 ± 1.00	1.68 ± 0.96	1.78 ± 1.14	0.838
Total−cholesterol	4.57 ± 1.20	4.63 ± 1.19	4.35 ± 1.22	0.152
LDL	3.16 ± 1.39	3.24 ± 1.43	2.83 ± 1.18	0.082
HDL	1.16 ± 0.47	1.17 ± 0.49	1.12 ± 0.40	0.670
Inflammatory markers White cell count, × 109/L	6.83 ± 1.70	6.78 ± 1.72	7.05 ± 1.61	0.222
CCTA acquisition parameters Radiation dose, DLP	411.58 ± 276.55	394.55 ± 228.22	481.14 ± 416.28	0.490
Tube voltage 70kv	134 (56.07)	105 (54.69)	29 (61.70)	0.481
80kv	89 (37.24)	76 (39.58)	13 (27.66)	0.178
90kv	10 (4.18)	7 (3.65)	3 (6.38)	0.665
110kv	7 (2.93)	5 (2.60)	2 (4.26)	0.905
Heart rate	74.10 ± 10.73	73.93 ± 10.17	74.77 ± 12.85	0.700
Systolic_pressure	133.71 ± 23.65	133.48 ± 24.08	134.64 ± 22.02	0.619
Diastolic_pressure	76.53 ± 10.38	76.48 ± 10.26	76.72 ± 10.97	0.888
Ultrasonic cardiogram LVDD	46.17 ± 4.64	45.73 ± 4.01	47.96 ± 6.35	0.025
LAD	35.08 ± 5.24	34.57 ± 3.81	37.17 ± 8.74	0.276
RAD	46.17 ± 4.64	45.73 ± 4.01	47.96 ± 6.35	0.025
RVDD	21.69 ± 2.33	21.52 ± 1.95	22.39 ± 3.41	0.118
EF	0.63 ± 0.07	0.64 ± 0.06	0.60 ± 0.09	<0.001
FS	0.50 ± 1.85	0.38 ± 0.07	0.95 ± 4.18	<0.001
EDV	110.74 ± 31.01	107.41 ± 26.50	124.32 ± 42.72	0.082
MVE	0.72 ± 0.18	0.71 ± 0.16	0.75 ± 0.22	0.548
MVA	0.89 ± 0.18	0.89 ± 0.17	0.90 ± 0.22	0.987
eGFR	108.14 ± 6.39	110.19 ± 4.63	99.77 ± 5.72	<0.001
Serum Creatinine	1.05 ± 0.24	0.99 ± 0.21	1.28 ± 0.21	<0.001
BUN	15.93 ± 2.93	15.50 ± 2.78	17.72 ± 3.02	<0.001

p-values were derived from the univariable association analysis between different variables; data are means with a statistical difference. p value reflected the differences between the MACE cohort and no MACE cohort. MACE, major adverse cardiac events; LDL, low-density lipoprotein; HDL, high-density lipoprotein; CCTA, coronary computed tomography angiography; DLP, dose-length product; BP blood pressure; BMI, body mass indexI; ACEI, angiotensin converting enzyme inhibitor; ARB, vasopressin II receptor blocker; LVDD, left atrium end diastolic diameter; LAD, left atrium diameter; RAD, right atrium diameter; RVDD, right ventricular end diastolic diameter; EF, ejection fraction; FS, fraction shorting; EDV, end-diastolic volume; MVE, mitral valve echogram; MVA, mitral valve area.

### 3.2 Feature selection and prediction model building

Radiomic features were extracted from regions of interest (ROIs) corresponding to the three main coronary arteries of patients, including morphological features (24), first-order histogram features (18), and higher-order texture features (51). Following standardization and redundancy reduction via Spearman correlation analysis, 11 radiomic features remained.

**Figure 2:**
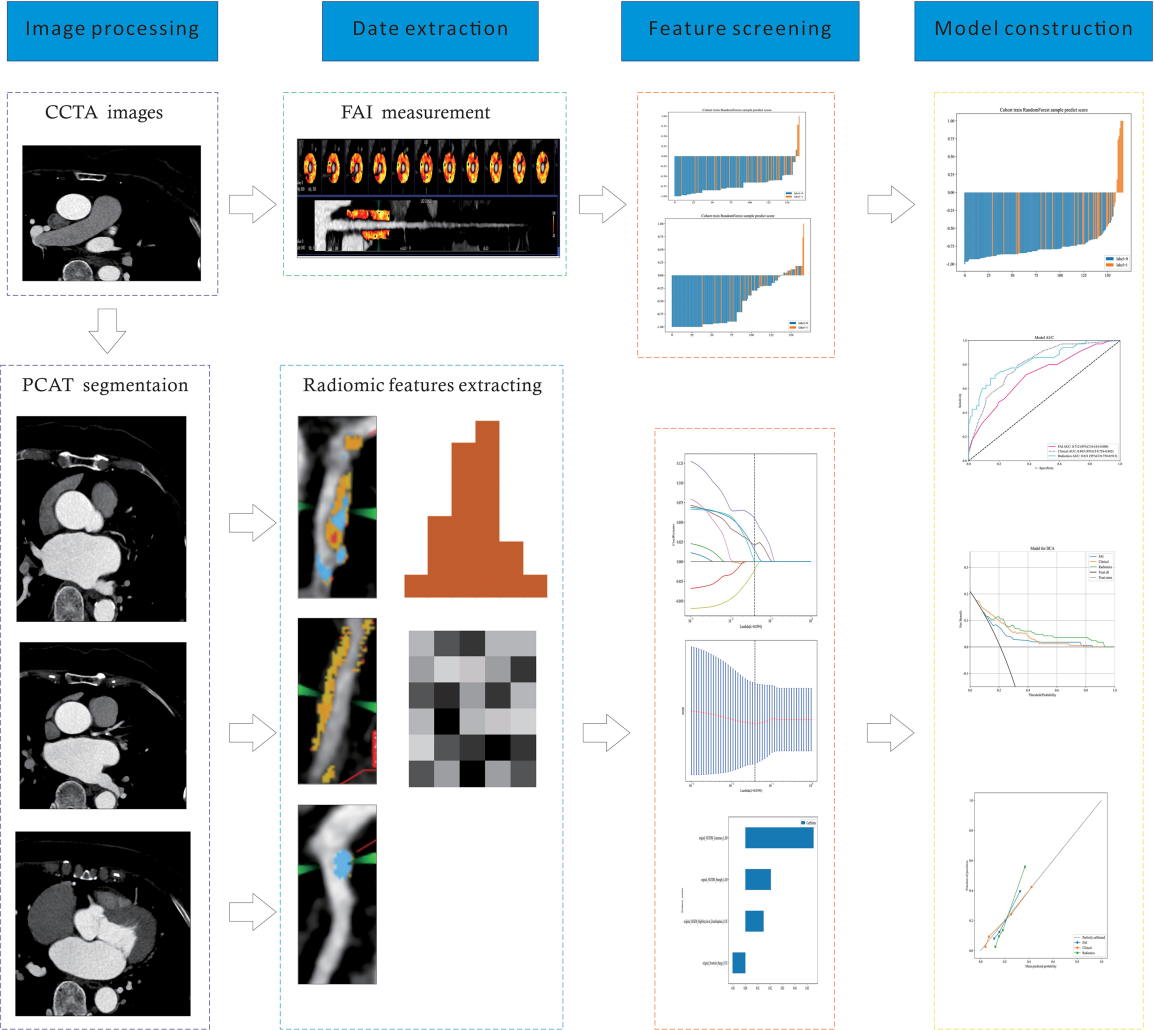
A flow chart of prediction model development process. CCTA, coronary computed tomography angiography; PCAT, pericoronary adipose tissue; FAI, fat attenuation index.

After final selection through LASSO regression, the four most relevant features were chosen for the construction of the radiomic feature model. Figure 2 depicts the selection process of the radiomic features in this study. All independent clinical predictors underwent univariate logistic regression analysis, and variables with *p* < 0.05 were included in subsequent analyses. A stepwise elimination approach was then used to construct a multivariate logistic regression analysis, ultimately identifying age as an independent predictor of major cardiovascular adverse events in patients with angina pectoris (*P* < 0.05). Based on this result, a clinical model was constructed using machine learning methods, with detailed results presented in Table 2. Lastly, the same methodology was applied to build the FAI model using the extracted fat attenuation index values.

**Table 2:** Logical regression analysis of the independent predictors.

Variables	Univariate regression analysis			Multivariate regression analysis		
	OR	95%CI	*P*	OR	95%CI	*P*
Age	1.009	1.004,1.013	0.003	1.009	1.004,1.013	0.002
Gender	1.019	0.918,1.131	–0.772	–	–	–
Body mass index	0.981	0.963,1.000	0.094	–	–	–
Hypertension	1.089	0.980,1.209	0.181	–	–	–
Diabetes	1.112	0.977,1.266	0.177	–	–	–
Smoking	1.072	0.963,1.194	0.288	–	–	–
Triglycerides	1.024	0.975,1.075	0.425	–	–	–
Total cholesterol	0.972	0.931,1.014	0.269	–	–	–
LDL cholesterol	0.978	0.939,1.018	0.356	–	–	–
HDL cholesterol	0.930	0.830,1.041	0.290	–	–	–
Antiplatelet	1.072	0.935,1.229	0.399	–	–	–
Statin	1.061	0.928,1.214	0.465	–	–	–
Beta-blocker	1.044	0.853,1.279	0.723	–	–	–
ACE-I or ARB	1.127	0.957,1.328	0.229	–	–	–
White cell count	1.022	0.989,1.057	0.280	-	–	–
Systolic BP	1.001	0.999,1.003	0.366	–	–	–
Diastolic BP	1.002	0.996,1.007	0.642	–	–	–
Heart rate	1.002	0.997,1.006	0.609	–	–	–
LVDD	1.019	1.008,1.030	0.006	0.995	0.979,1.011	0.581
LAD	1.019	1.010,1.028	0.001	1.006	0.995,1.018	0.354
RAD	1.021	1.009,1.031	0.002	1.008	0.994,1.022	0.346
RVDD	1.030	1.009,1.051	0.021	1.008	0.982,1.035	0.61
EF	0.171	0.086,0.343	0.000	0.382	0.143,1.019	0.107
FS	0.299	0.146,0.611	0.006	0.753	0.326,1.744	0.578
EDV	1.003	1.002,1.005	0.001	1.002	1.000,1.004	0.061
MVE	1.178	0.875,1.582	0.363	–	–	–
MVA	1.233	0.933,1.629	0.216	–	–	–

LDL, low-density lipoprotein; HDL, high-density lipoprotein; CCTA, coronary computed tomography angiography; BP, blood pressure; BMI, body mass indexI; ACEI, angiotensin converting enzyme inhibitor; ARB, vasopressin II receptor blocker; LVDD, left atrium end diastolic diameter; LAD, left atrium diameter; RAD, right atrium diameter; RVDD, right ventricular end diastolic diameter; EF, ejection fraction; FS, fraction shorting; EDV, end-diastolic volume; MVE, mitral valve echogram; MVA, mitral valve area.

### 3.3 Efficacy evaluation

**Figure 3:**
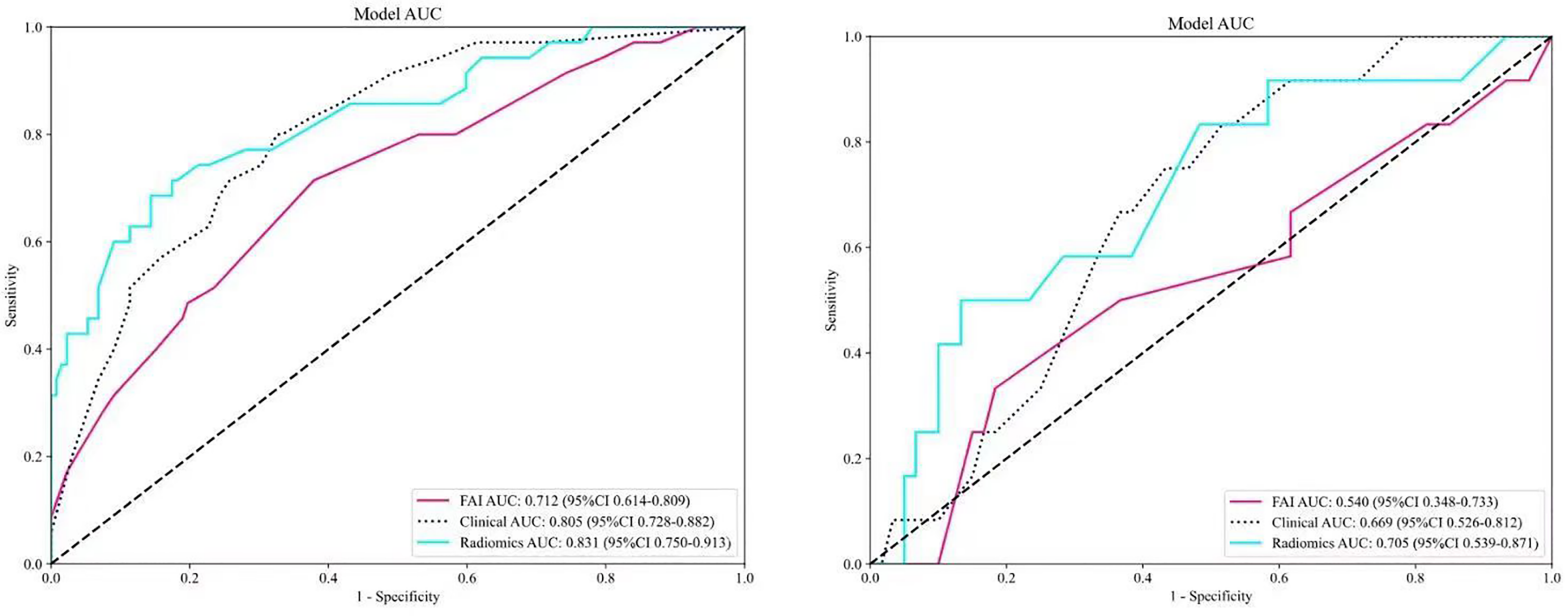
Comparison of receiver operating characteristic (ROC) curves for the FAI model (red lines), clinical model (blue dotted lines) and radiomics model (blue solid lines).

**Figure 4:**
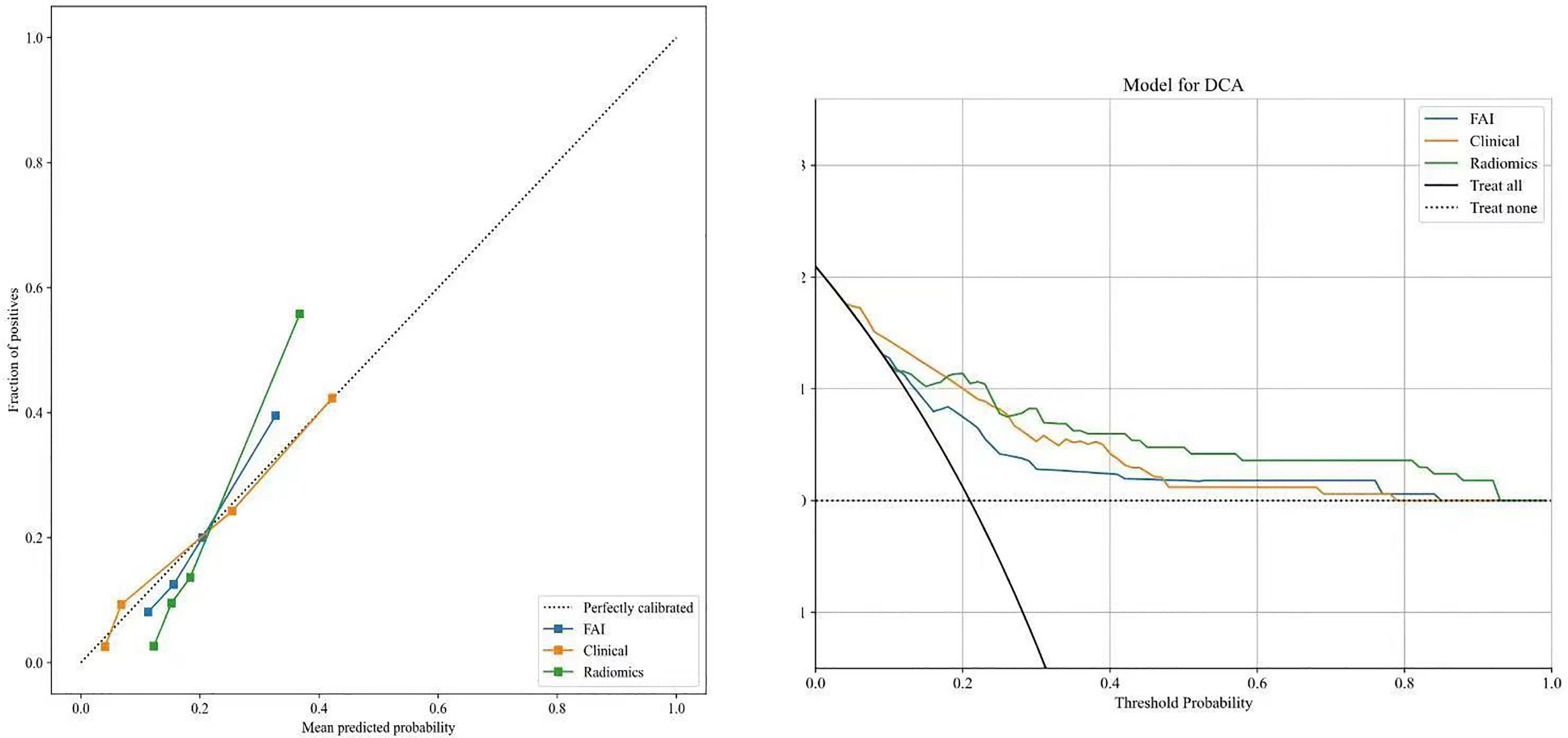
Decision curve analysis and calibration curves of the FAI model (blue line), clinical model(orange line) and radiomics model(green line).

ROC curves for the three models were plotted in both the training and validation sets to illustrate their predictive performances (see Figure 3). The radiomics model showed superior efficacy with AUCs of 0.83 [95% CI: 0.750–0.913] and 0.71 [95% CI: 0.539–0.871], compared to the clinical feature model (AUC = 0.81 [95%CI: 0.728–0.882], AUC = 0.67 [95%CI: 0.526–0.812]) and the FAI model (AUC = 0.71 [95%CI: 0.614–0.809], AUC = 0.54 [95%CI: 0.348–0.733]). The predictive abilities of the three models were quantified by measuring accuracy, specificity, sensitivity, positive predictive value, and negative predictive value (Table 3). Decision curves demonstrated the clinical utility of the three predictive models by comparing net benefits at different threshold probabilities in the dataset, with results indicating that the net benefit of predicting MACE in angina pectoris was greater with the radiomics model than with the other models (see Figure 4). Calibration curves for the three predictive models showed good agreement between predicted and observed probabilities of adverse events in angina pectoris (see Figure 4).

**Table 3:** Predictive ability of all models.

Model	Training cohort						Validation cohort					
	AUC (95% CI)	SPE	SEN	ACC	PPV	NPV	AUC (95% CI)	SPE	SEN	ACC	PPV	NPV
FAI	0.71 (0.61–0.81)	0.77	0.51	0.71	0.37	0.86	0.54 (0.35–0.73)	0.83	0.25	0.74	0.23	0.85
Clinical	0.81 (0.73–0.88)	0.70	0.74	0.71	0.39	0.91	0.67 (0.53–0.81)	0.62	0.67	0.63	0.26	0.90
Radiomics	0.83 (0.75–0.91)	0.86	0.63	0.81	0.54	0.90	0.71 (0.54–0.87)	0.87	0.42	0.79	0.39	0.88

FAI, fat attenuation index; AUC, area under curve; 95% CI, 95% confidence interval; SPE, specificity; SEN, sensitivity; ACC, accuracy; PPV, positive predictive value; NPV, negative predictive valve.

## 4 Discussion

With the deepening research on coronary artery disease, a variety of effective consensus treatment methods have emerged. Conservative treatment remains a baseline strategy aimed at symptom management, preventing the progression of disease, and averting adverse events, particularly myocardial infarction. Invasive treatments such as percutaneous coronary intervention (PCI) or coronary artery bypass grafting (CABG) can complement conservative measures (1, 23). In our study, patients with angina were initially managed with conservative treatments, including anti-anginal therapy, aimed at symptom relief and prevention of disease progression. Modifications to anti-anginal therapy were made on a case-by-case basis during the follow-up period, depending on changes in symptomatology, although detailed tracking of therapy modifications was not a primary focus of this study. Invasive treatments, such as PCI or CABG, were performed for a subset of patients based on baseline CCTA findings. These procedures were undertaken when significant coronary stenosis or high-risk plaque features were identified, necessitating further intervention to mitigate the risk of MACE. During the follow-up period, 15% of patients underwent PCI and 5% underwent CABG. The general discussion of treatment strategies in this manuscript is based on established literature on coronary artery disease management. Our study reflects real-world clinical decision-making, where a combination of conservative and invasive strategies was employed depending on the individual patient's risk profile and clinical presentation. However, numerous patients still suffer serious cardiovascular adverse events, and the specific risk factors and likelihood of occurrence are not entirely clear. Therefore, researchers are constructing predictive models by exploring the factors related to major cardiovascular adverse events in ST-elevation myocardial infarction to further assess risks and guide treatment (24). An increasing number of patients presenting with angina pectoris for consultation and treatment also frequently experience severe and irreversible cerebrovascular accidents during the progression of their disease (2, 3). However, current research into the major adverse cardiovascular events associated with angina pectoris is not in-depth, and early identification of high-risk factors for MACE events in patients with angina pectoris, constructing evaluation systems suitable for specific populations, and managing individuals are crucial. Consequently, this study attempts to optimize individualized risk prediction through the establishment of a radiomics model, offering a novel spatial perspective. Clinically, CCTA serves as a frontline diagnostic tool for assessing coronary artery disease patients and is an excellent means to determine arterial plaque burden and disease progression. PCAT is directly adjacent to the coronary vessels and interacts with the formation and progression of atherosclerotic plaques in a bidirectional manner. PCAT can sensitively respond to vascular inflammation, causing changes in the size, shape, and distribution of adipocytes in pericoronary adipose tissue (13), and FAI can quantify these PCAT changes. Therefore, observers use CCTA to quantitatively assess coronary lumen stenosis, coronary plaques, and high-risk plaque characteristics, while also qualitatively evaluating low-attenuation plaques, napkin-ring signs, and spotty calcifications (20). CCTA's recognition of features such as high-risk plaques is applicable to plaques forming later in the disease process, while early inflammation, plaque structure, and luminal microenvironment analysis require the introduction of plaque radiomic characterization for assessment (13, 25). Thus, the FAI model in this study indeed exhibited a certain gap in predictive capability compared to the emerging radiomics model. Age is recognized as a primary risk factor for the development of atherosclerotic cardiovascular diseases and coronary heart disease. Studies have shown that age correlates more strongly with the risk of cardiovascular disease events in men than any other factor, and in women, it is second only to hypertension (26, 27). To further analyze the factor of age, scholars have found that patients under the age of 65 have a higher incidence of chest pain, while those over 65 experience a reduction in chest pain (28). However, mortality rates also increase with age. This leads patients to often overlook the high risk associated with age due to the alleviation of chest pain symptoms. When investigating the underlying reasons, the telomere hypothesis has been proposed. Telomere shortening is considered a marker of the aging process and can lead to arterial atherosclerosis, thereby causing cardiovascular disease (29). As age increases, telomeres become progressively shorter, but it appears that patients with coronary heart disease who have a more balanced nutritional diet may have elongated telomeres (29). Through the analysis and selection of clinical factor correlations, this study also confirmed that age has the closest relationship with the occurrence of adverse cardiovascular events in patients with angina pectoris, and the clinical model established as such also exhibited good predictive capacity. Radiomics is a rapidly developing new technology that extracts and analyzes imaging features by segmenting images of the ROI, considering the complex spatial relationships between voxels (12, 13). It can also reflect more persistent changes in the perivascular space caused by vascular inflammation, such as fibrosis and neovascularization (15). In this study, images from the region of interest in coronary CT were segmented and extracted. After analyzing and filtering the PCAT from the proximal parts of the three main coronary arteries, four most robust radiomic features were obtained. These features were used to construct the radiomics model, demonstrating its good efficacy in predicting major adverse cardiovascular events in patients with angina pectoris. Although we did not directly link radiomic features with histopathological analysis, precluding determination of their specific correlation with histopathological changes, radiomic features are big data parameters derived from pixel distribution, serving as an interpretation of the structure underlying the images. They are also an important supplement to data providing information about the imaging phenotype, potentially including a wealth of information (30, 31).

Our study also has some limitations. On one hand, the sample size included in the study is relatively limited, with a correspondingly low incidence rate of events. The presence of a low prevalence binary predictive variable (MACEs negative or positive) may lead to issues of complete or quasi-complete separation in logistic regression (32). Therefore, larger populations and external validations are needed for future studies. On the other hand, all patients’ imaging pictures were taken from the same CT scanner and settings. Since image acquisition, reconstruction, and analysis can affect the reproducibility of imaging features, no studies have explored how these settings may affect radiomic parameters, and our model will need to be validated in different CT scanners in the future (33–35). Renal function, as measured by eGFR, serum creatinine, and BUN, was found to differ between the MACE and non-MACE groups. Patients in the MACE group exhibited lower eGFR and higher serum creatinine and BUN levels, consistent with the known association between impaired renal function and increased cardiovascular risk. However, despite these differences, the inclusion of renal function parameters in our predictive model did not significantly enhance its performance in predicting MACE. This may be due to the relatively preserved renal function in the majority of our cohort, or the small sample size. Nevertheless, renal impairment remains an important risk factor for cardiovascular events, and future studies with larger populations may explore this relationship in greater detail.

## 5 Conclusion

In summary, the PCAT radiomics model based on coronary CT can effectively predict the occurrence of major adverse cardiovascular events in patients with angina pectoris. This user-friendly tool can help clinicians optimize risk stratification and management of individual patients.

## Data Availability

The original contributions presented in the study are included in the article/Supplementary Material, further inquiries can be directed to the corresponding author.
